# Supplementary material for: Relationships between Heavy Metal Concentrations in Greater Celandine (Chelidonium majus L.) Tissues and Soil in Urban Parks
Source: Int J Environ Res Public Health. 2023 Feb 22;20(5):3887. doi: 10.3390/ijerph20053887 (PMC10002234; doi:10.3390/ijerph20053887)
Supplement: Supplementary file 1 [file ijerph-20-03887-s001.zip › ijerph-2243698-supplementary.pdf]

Table S1. Spearman's rank correlation coefficient in terms of soil physicochemical properties.

	H2O	KCl	Loss_on_ignition	Corg	Nt	CN	Mgavail	K	Pavail	Pt	Hh	Al3	H
H2O	1	.943**	-0.43	-0.09	-0.77	0.54	0.14	0.31	-0.14	-0.2	-.943**	0.09	-.943**
KCl	.943**	1	-0.14	0.14	-0.66	0.6	0.26	0.43	0.09	0.09	-.886*	0.32	-.886*
Loss_on_ignition	-0.43	-0.14	1	0.37	0.49	-0.09	0.26	0.43	0.77	0.77	0.37	0.55	0.37
Corg	-0.09	0.14	0.37	1	0.03	0.6	0.09	-0.09	0.09	0.26	0.14	.841*	0.14
Nt	-0.77	-0.66	0.49	0.03	1	-0.77	0.49	0.2	0.6	0.6	.886*	-0.03	.886*
CN	0.54	0.6	-0.09	0.6	-0.77	1	-0.31	-0.14	-0.37	-0.31	-0.6	0.61	-0.6
Mgavail	0.14	0.26	0.26	0.09	0.49	-0.31	1	.829*	0.77	0.6	0.14	0.32	0.14
K	0.31	0.43	0.43	-0.09	0.2	-0.14	.829*	1	.829*	0.54	-0.14	0.38	-0.14
Pavail	-0.14	0.09	0.77	0.09	0.6	-0.37	0.77	.829*	1	.886*	0.26	0.38	0.26
Pt	-0.2	0.09	0.77	0.26	0.6	-0.31	0.6	0.54	.886*	1	0.26	0.32	0.26
Hh	-.943**	-.886*	0.37	0.14	.886*	-0.6	0.14	-0.14	0.26	0.26	1	0	1.000**
Al3	0.09	0.32	0.55	.841*	-0.03	0.61	0.32	0.38	0.38	0.32	0	1	0
H	-.943**	-.886*	0.37	0.14	.886*	-0.6	0.14	-0.14	0.26	0.26	1.000**	0	1

* The correlation is significant at the level of 0.05 (two-sided).

**.The correlation is significant at the level of 0.01 (two-sided).

Table S2. Spearman rank correlation coefficient between soil elements and rhizomes.

	Pb	Cd	Zn	Mn	Fe	Cu	Ni	Cr	Hg	As	Pb_rh.	Cd_rh.	Zn_rh.	Mn_rh.	Fe_rh.	Cu_rh.	Ni_rh.	Crh._rh.	Hg_rh.	As_rh.
Pb	1	.829*	.829*	.943**	.829*	.943**	.829*	0.714	.886*	.943**	.943**	0.657	0.486	.314	.943**	.429	.771	0.657	.754	.829*
Cd	.829*	1	1.000**	.943**	0.543	0.657	.829*	0.429	0.6	0.771	.943**	0.429	0.143	.257	.771	.143	.486	.371	0.29	.886*
Zn	.829*	1.000**	1	.943**	0.543	0.657	.829*	0.429	0.6	0.771	.943**	0.429	0.143	.257	.771	.143	.486	.371	.290	.886*
Mn	.943**	.943**	.943**	1	0.657	.829*	0.771	0.6	0.714	.829*	1.000**	0.486	0.257	.371	.886*	.314	.600	.486	.551	.943**
Fe	.829*	0.543	0.543	0.657	1	.886*	0.771	0.429	.943**	.886*	0.657	.943**	.886*	.143	.886*	.714	.600	.486	.754	.543
Cu	.943**	0.657	0.657	.829*	.886*	1	0.714	0.6	.943**	.886*	.829*	0.771	0.657	.143	.886*	.657	.714	.543	.899*	.771
Ni	.829*	.829*	.829*	0.771	0.771	0.714	1	0.486	.829*	.943**	0.771	0.714	0.486	0.029	0.771	0.2	0.714	0.6	0.435	0.6
Cr	0.714	0.429	0.429	0.6	0.429	0.6	0.486	1	0.543	0.657	0.6	0.143	0.086	0.486	0.6	-0.086	.886*	.943**	0.638	0.371
Hg	.886*	0.6	0.6	0.714	.943**	.943**	.829*	0.543	1	.943**	0.714	.886*	0.771	-0.029	.829*	0.6	0.771	0.6	.841*	0.6
As	.943**	0.771	0.771	.829*	.886*	.886*	.943**	0.657	.943**	1	.829*	0.771	0.6	0.143	.886*	0.371	.829*	0.714	0.696	0.657
Pb_rh.	.943**	.943**	.943**	1.000**	0.657	.829*	0.771	0.6	0.714	.829*	1	0.486	0.257	0.371	.886*	0.314	0.6	0.486	0.551	.943**
Cd_rh.	0.657	0.429	0.429	0.486	.943**	0.771	0.714	0.143	.886*	0.771	0.486	1	.943**	-0.143	0.714	0.771	0.429	0.257	0.638	0.429
Zn_rh.	0.486	0.143	0.143	0.257	.886*	0.657	0.486	0.086	0.771	0.6	0.257	.943**	1	-0.086	0.6	.829*	0.314	0.2	0.638	0.2
Mn_rh.	0.314	0.257	0.257	0.371	0.143	0.143	0.029	0.486	-0.029	0.143	0.371	-0.143	-0.086	1	0.486	-0.029	0.086	0.314	0.058	0.257
Fe_rh.	.943**	0.771	0.771	.886*	.886*	.886*	0.771	0.6	.829*	.886*	.886*	0.714	0.6	0.486	1	0.543	0.6	0.543	0.667	0.771
Cu_rh.	0.429	0.143	0.143	0.314	0.714	0.657	0.2	-0.086	0.6	0.371	0.314	0.771	.829*	-0.029	0.543	1	0.029	-0.143	0.638	0.429
Ni_rh.	0.771	0.486	0.486	0.6	0.6	0.714	0.714	.886*	0.771	.829*	0.6	0.429	0.314	0.086	0.6	0.029	1	.943**	0.725	0.371
Crh._rh.	0.657	0.371	0.371	0.486	0.486	0.543	0.6	.943**	0.6	0.714	0.486	0.257	0.2	0.314	0.543	-0.143	.943**	1	0.58	0.2
Hg_rh.	0.754	0.29	0.29	0.551	0.754	.899*	0.435	0.638	.841*	0.696	0.551	0.638	0.638	0.058	0.667	0.638	0.725	0.58	1	0.493
As_rh.	.829*	.886*	.886*	.943**	0.543	0.771	0.6	0.371	0.6	0.657	.943**	0.429	0.2	0.257	0.771	0.429	0.371	0.2	0.493	1

* The correlation is significant at the level of 0.05 (two-sided).

**.The correlation is significant at the level of 0.01 (two-sided).

*_rh.- rhizome

Table S3. Spearman rank correlation coefficient between elements in soil and stem.

	Pb	Cd	Zn	Mn	Fe	Cu	Ni	Cr	Hg	As	Pb_st	Cd_st	Zn_st	Mn_st	Fe_st	Cu_st	Ni_st	Cr_st	Hg_st	Ast_st
Pb	1	.829*	.829*	.943**	.829*	.943**	.829*	0.714	.886*	.943**	0.257	0.771	0.657	0.232	0.471	0.6	0.657	0.429	0.754	-0.525
Cd	.829*	1	1.000**	.943**	0.543	0.657	.829*	0.429	0.6	0.771	0.6	0.771	0.429	0.29	0.441	0.6	0.429	0.257	0.348	-0.185
Zn	.829*	1.000**	1	.943**	0.543	0.657	.829*	0.429	0.6	0.771	0.6	0.771	0.429	0.29	0.441	0.6	0.429	0.257	0.348	-0.185
Mn	.943**	.943**	.943**	1	0.657	.829*	0.771	0.6	0.714	.829*	0.486	0.714	0.486	0.377	0.559	0.657	0.6	0.371	0.551	-0.309
Fe	.829*	0.543	0.543	0.657	1	.886*	0.771	0.429	.943**	.886*	-0.257	0.6	.943**	-0.116	0.235	0.543	0.257	0.029	0.638	-.833*
Cu	.943**	0.657	0.657	.829*	.886*	1	0.714	0.6	.943**	.886*	0.086	0.6	0.771	0.029	0.588	0.714	0.6	0.371	.812*	-0.741
Ni	.829*	.829*	.829*	0.771	0.771	0.714	1	0.486	.829*	.943**	0.257	.943**	0.714	-0.087	0.147	0.429	0.371	0.257	0.551	-0.494
Cr	0.714	0.429	0.429	0.6	0.429	0.6	0.486	1	0.543	0.657	0.2	0.657	0.143	0.464	0.088	-0.029	.886*	0.771	.812*	-0.093
Hg	.886*	0.6	0.6	0.714	.943**	.943**	.829*	0.543	1	.943**	-0.029	0.714	.886*	-0.203	0.383	0.6	0.486	0.314	.812*	-.833*
As	.943**	0.771	0.771	.829*	.886*	.886*	.943**	0.657	.943**	1	0.143	.886*	0.771	0	0.265	0.486	0.543	0.371	0.754	-0.617
Pb_st	0.257	0.6	0.6	0.486	-0.257	0.086	0.257	0.2	-0.029	0.143	1	0.371	-0.314	0.232	0.5	0.314	0.486	0.543	0.087	0.37
Cd_st	0.771	0.771	0.771	0.714	0.6	0.6	.943**	0.657	0.714	.886*	0.371	1	0.486	0.029	0.029	0.2	0.543	0.486	0.609	-0.278
Zn_st	0.657	0.429	0.429	0.486	.943**	0.771	0.714	0.143	.886*	0.771	-0.314	0.486	1	-0.406	0.235	0.6	0.029	-0.143	0.493	-.926**
Mn_st	0.232	0.29	0.29	0.377	-0.116	0.029	-0.087	0.464	-0.203	0	0.232	0.029	-0.406	1	-0.015	-0.174	0.319	0.145	-0.059	0.564
Fe_st	0.471	0.441	0.441	0.559	0.235	0.588	0.147	0.088	0.383	0.265	0.5	0.029	0.235	-0.015	1	.883*	0.441	0.324	0.373	-0.365
Cu_st	0.6	0.6	0.6	0.657	0.543	0.714	0.429	-0.029	0.6	0.486	0.314	0.2	0.6	-0.174	.883*	1	0.2	0.029	0.319	-0.617
Ni_st	0.657	0.429	0.429	0.6	0.257	0.6	0.371	.886*	0.486	0.543	0.486	0.543	0.029	0.319	0.441	0.2	1	.943**	.841*	-0.093
Cr_st	0.429	0.257	0.257	0.371	0.029	0.371	0.257	0.771	0.314	0.371	0.543	0.486	-0.143	0.145	0.324	0.029	.943**	1	0.754	0.031
Hg_st	0.754	0.348	0.348	0.551	0.638	.812*	0.551	.812*	.812*	0.754	0.087	0.609	0.493	-0.059	0.373	0.319	.841*	0.754	1	-0.579
Ast_st	-0.525	-0.185	-0.185	-0.309	-.833*	-0.741	-0.494	-0.093	-.833*	-0.617	0.37	-0.278	-.926**	0.564	-0.365	-0.617	-0.093	0.031	-0.579	1

*The correlation is significant at the level of 0.05 (two-sided); **The correlation is significant at the level of 0.01 (two-sided).

*_st.- stem

Table S4. Spearman rank correlation coefficient between elements in soil and leaves.

	Pb	Cd	Zn	Mn	Fe	Cu	Ni	Cr	Hg	As	Pb_le	Cd_le	Zn_le	Mn_le	Fe_le	Cu_le	Ni_le	Cr_le	Hg_le	As_le
Pb	1	.829*	.829*	.943**	.829*	.943**	.829*	0.714	.886*	.943**	0.6	0.371	0.657	0.714	0.53	.943**	0.771	0.429	0.406	0.696
Cd	.829*	1	1.000**	.943**	0.543	0.657	.829*	0.429	0.6	0.771	.829*	0.6	0.714	0.714	0.677	0.771	0.6	0.2	0.232	.899*
Zn	.829*	1.000**	1	.943**	0.543	0.657	.829*	0.429	0.6	0.771	.829*	0.6	0.714	0.714	0.677	0.771	0.6	0.2	0.232	.899*
Mn	.943**	.943**	.943**	1	0.657	.829*	0.771	0.6	0.714	.829*	0.771	0.429	0.6	0.771	0.736	.886*	0.657	0.257	0.319	.841*
Fe	.829*	0.543	0.543	0.657	1	.886*	0.771	0.429	.943**	.886*	0.086	0.086	0.714	0.429	0.059	.886*	0.6	0.257	0.058	0.232
Cu	.943**	0.657	0.657	.829*	.886*	1	0.714	0.6	.943**	.886*	0.371	0.086	0.6	0.486	0.441	.886*	0.6	0.314	0.348	0.464
Ni	.829*	.829*	.829*	0.771	0.771	0.714	1	0.486	.829*	.943**	0.543	0.657	.943**	0.543	0.206	0.771	0.771	0.486	0.319	0.667
Cr	0.714	0.429	0.429	0.6	0.429	0.6	0.486	1	0.543	0.657	0.543	0.486	0.2	0.714	0.324	0.6	.886*	.829*	0.754	0.58
Hg	.886*	0.6	0.6	0.714	.943**	.943**	.829*	0.543	1	.943**	0.257	0.2	0.771	0.371	0.177	.829*	0.657	0.429	0.348	0.377
As	.943**	0.771	0.771	.829*	.886*	.886*	.943**	0.657	.943**	1	0.486	0.486	.829*	0.6	0.265	.886*	.829*	0.543	0.406	0.609
Pb_le	0.6	.829*	.829*	0.771	0.086	0.371	0.543	0.543	0.257	0.486	1	0.714	0.371	0.657	0.794	0.429	0.543	0.371	0.551	.986**
Cd_le	0.371	0.6	0.6	0.429	0.086	0.086	0.657	0.486	0.2	0.486	0.714	1	0.543	0.543	0.147	0.257	0.714	0.657	0.493	0.754
Zn_le	0.657	0.714	0.714	0.6	0.714	0.6	.943**	0.2	0.771	.829*	0.371	0.543	1	0.257	0.059	0.6	0.543	0.314	0.174	0.493
Mn_le	0.714	0.714	0.714	0.771	0.429	0.486	0.543	0.714	0.371	0.6	0.657	0.543	0.257	1	0.5	0.771	0.771	0.371	0.203	0.725
Fe_le	0.53	0.677	0.677	0.736	0.059	0.441	0.206	0.324	0.177	0.265	0.794	0.147	0.059	0.5	1	0.412	0.147	-0.088	0.284	0.746
Cu_le	.943**	0.771	0.771	.886*	.886*	.886*	0.771	0.6	.829*	.886*	0.429	0.257	0.6	0.771	0.412	1	0.714	0.257	0.116	0.551
Ni_le	0.771	0.6	0.6	0.657	0.6	0.6	0.771	.886*	0.657	.829*	0.543	0.714	0.543	0.771	0.147	0.714	1	.829*	0.58	0.638
Cr_le	0.429	0.2	0.2	0.257	0.257	0.314	0.486	.829*	0.429	0.543	0.371	0.657	0.314	0.371	-0.088	0.257	.829*	1	.841*	0.406
Hg_le	0.406	0.232	0.232	0.319	0.058	0.348	0.319	0.754	0.348	0.406	0.551	0.493	0.174	0.203	0.284	0.116	0.58	.841*	1	0.515
As_le	0.696	.899*	.899*	.841*	0.232	0.464	0.667	0.58	0.377	0.609	.986**	0.754	0.493	0.725	0.746	0.551	0.638	0.406	0.515	1

*The correlation is significant at the level of 0.05 (two-sided).e).

** The correlation is significant at the level of 0.01 (two-sided).

*_le.- leaves
